# Plant extract preparation and green synthesis of silver nanoparticles using *Swertia chirata*: Characterization and antimicrobial activity against selected human pathogens

**DOI:** 10.1016/j.heliyon.2024.e28038

**Published:** 2024-03-15

**Authors:** Muhammad Adnan Shereen, Aftab Ahmad, Hashir Khan, Sadia Mehmood Satti, Abeer Kazmi, Nadia Bashir, Muhammad Shehroz, Shahid Hussain, Muhammad Ilyas, M. Ijaz Khan, Hatoon A. Niyazi, Ferjeni Zouidi

**Affiliations:** b Department of Microbiology, Kohsar University Murree, Murree, 47150, Pakistan; c Alpha Genomics (Pvt), PWD Society, Islamabad, Punjab, Pakistan; d The State Key Laboratory of Freshwater Ecology and Biotechnology, The Key Laboratory of Aquatic Biodiversity and Conservation of Chinese Academy of Sciences, Institute of Hydrobiology, Chinese Academy of Sciences, Wuhan, 430072, Hubei, PR China; e University of Chinese Academy of Sciences, Beijing, 100049, PR China; f Department of Microbiology, College of Life Sciences, Wuhan University, 430072, Wuhan, PR China; g Department of Bioinformatics, Kohsar University Murree, Murree, 47150, Pakistan; h Department of Biotechnology, Kohsar University Murree, Murree, 47150, Pakistan; i Department of Botany, Kohsar University Murree, Murree, 47150, Pakistan; j Department of Mechanical Engineering, Lebanese American University, Kraytem, 1102-2801, Beirut, Lebanon; k Department of Clinical Microbiology and Immunology, Faculty of Medicine, King Abdulaziz University Jeddah, 21589, Saudi Arabia; l Faculty of Science and Arts, Muhayil Asser, King Khalid University, Saudi Arabia

**Keywords:** Green synthesis, Silver nanoparticles, *Swertia chirata*, Antimicrobial activity, Nanotechnology

## Abstract

Herbal medicinal plants have been used for centuries in traditional medicine, and it is interesting to see how modern research has identified the active compounds responsible for their therapeutic effects. The green synthesis of silver nanoparticles using herbal medicinal plants, such as *Swertia chirata*, is particularly noteworthy due to its antimicrobial properties. In the current study, the *Swertia chirata* plant was collected for the first time from the region of Murree, Punjab, Pakistan. After collection, extracts were prepared in different solvents (ethanol, methanol, chloroform, and distilled water), and silver nanoparticles were synthesized by reducing silver nitrate (AgNO_3_). The UV–visible spectrophotometer, SEM, and EDX were used to characterize the synthesized nanoparticles in terms of their size and shape. The phytochemical analysis of crude extract was performed to determine the presence of different kinds of phytochemicals. The antibacterial activity of plant extracts and the silver nanoparticles were then assessed using the agar well diffusion method against various pathogenic bacteria. The results showed that the plant contains several phytochemicals with remarkable antioxidant potential. The antibacterial analysis revealed that silver nanoparticles and the plant extracts exhibited a significant zone of inhibition against human pathogenic bacteria (*Escherichia coli*, *S. capitis*, *B. subtilis,* and *Pseudomonas aeruginosa*) as compared to the cefixime and norfloxacin. This implies that the nanoparticles have the potential to be used in nano-medicine applications, such as drug delivery systems, as well as for their antibacterial, antifungal, and antiviral activities. Additionally, the development and application of materials and technologies at the nanometer scale opens possibilities for the creation of novel drugs and therapies. Overall, the study highlights the promising potential of herbal medicinal plants found in Murree, Punjab, Pakistan, and green-synthesized silver nanoparticles in various fields of medicine and nanotechnology.

## 1 Introduction

Anti-microbial resistance (AMR) is a growing global health concern due to the development of drug-resistant bacteria due to the widespread use of antibiotics [1]. This has resulted in an urgent need for the development of new and effective antimicrobial agents. Medicinal plants have long been used in traditional medicine to treat various ailments, including bacterial infections [2]. Numerous studies have reported the antibacterial activity of plant extracts against a wide range of bacteria [3]. These plant-based antimicrobial agents offer several advantages over synthetic antibiotics, including a lower toxicity profile, fewer side effects, and a diverse range of bioactive compounds that can target different aspects of bacterial growth and survival, making it difficult for bacteria to develop resistance [4].

Plant extracts are known to contain various bioactive compounds, such as alkaloids, flavonoids, tannins, and phenolic compounds [5], which have been shown to possess antimicrobial activity. However, the efficacy of plant-based antibiotics is limited due to factors such as poor solubility, bioavailability, and stability [6]. To address these limitations, researchers have turned to the use of nanoparticles, which possess unique physical and chemical properties that make them promising candidates for antimicrobial applications [7]. Silver nanoparticles, in particular, have been extensively studied for their broad-spectrum antimicrobial activity against both gram-positive and gram-negative bacteria [8].

*Swertia chirata* is a species of the Gentianaceae family. Roxburgh specified *Gentiana chyrayta* for the first time in 1814. *Swertia chirata* is a critically endangered medicinal plant that grows at high elevations in the sub-temperate Himalayas between 1200 and 2100 m heights from Kashmir to Bhutan [9,10]. The extensive list of medical applications includes the treatment of melancholia, chronic fever, hypertension, malaria, scanty urine, anemia, ulcers, bronchial asthma, epilepsy, hepatotoxic disorders, hepatitis, worms, gastritis, skin diseases, dyspepsia, constipation, and some types of mental disorders, as well as diabetes, blood purification, secretion of bile [11,12]. *Swertia chirata* extracts recently demonstrated anti-hepatitis B virus (anti-HBV) properties [13,14]. Decoctions of this species have historically been used as cardio protectants, anthelmintics, hepatoprotectives, hypoglycemics, antimalarials, antifungals, antibacterials, cardiostimulants, antifatigue, anti-inflammatory, antiaging, and antidiarrheal [12,15,16]. It also helps to reduce blood pressure and sugar levels [17]. *Swertia chirata* extract is found in various amounts in herbal preparations such as Ayush-64, Diabecon, Mensturyl syrup, and Melicon V ointment [18]. Traditional Chinese Medicine (TCM) significantly reduced the SARS-Cov-2 infection in China during the start of the pandemic. *Swertia chirata* was the key plant extract in all the TCMs utilized against COVID-19 [[19], [20], [21], [22]].

Several studies have investigated the antibacterial activity of *Swertia chirata* extracts against various bacterial strains, including *Staphylococcus aureus*, *Escherichia coli*, and *Pseudomonas aeruginosa* [23]. However, there is limited information on the antibacterial activity of silver nanoparticles synthesized from *Swertia chirata* harvested from the forests of Murree, Punjab, Pakistan. Therefore, this study aims to ascertain if Swertia chirata plant extracts and silver nanoparticles produced in various solvents have any antibacterial effects on gram-positive and gram-negative bacteria. The findings of this study could provide insights into the potential use of plant-based antibiotics and nanoparticles for the treatment of bacterial infections.

## 2 Materials and methods

### 2.1 Collection of plant material

**Fig. 1:**
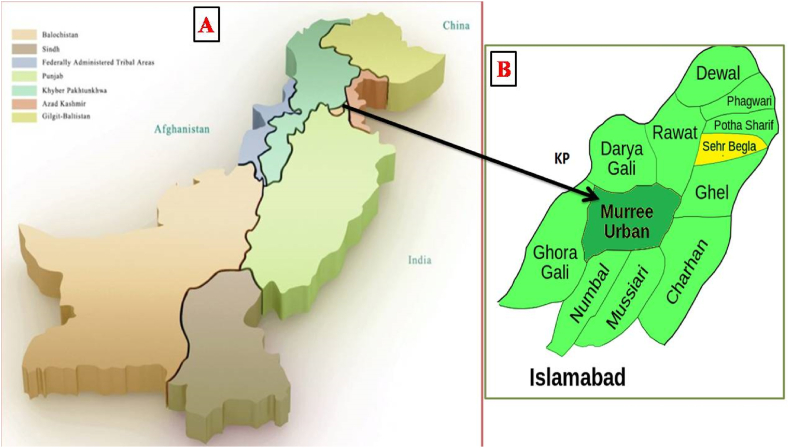
Map representing the site from where the samples of *Swertia chirata* were collected. (A) Map of Pakistan, (B) location of Murree.

The *Swertia chirata* plant was for the first time identified and collected from the forest of Murree, Punjab, Pakistan [Fig. 1(A and B)]. This study was conducted at the Microbiology lab, Department of Microbiology, Kohsar University Murree.

### 2.2 Processing of sample

To prepare the plant sample for analysis, the leaves and stems were isolated and rinsed with sterile distilled water to eliminate any debris or unwanted substances. Once cleaned, the leaves and stems were dried in the dry air oven at 60 °C for 4 h and finely ground into powder form [24]. The resulting powder was then securely kept in a sterile container for further analysis.

### 2.3 Preparation of plant extracts

**Fig. 2:**
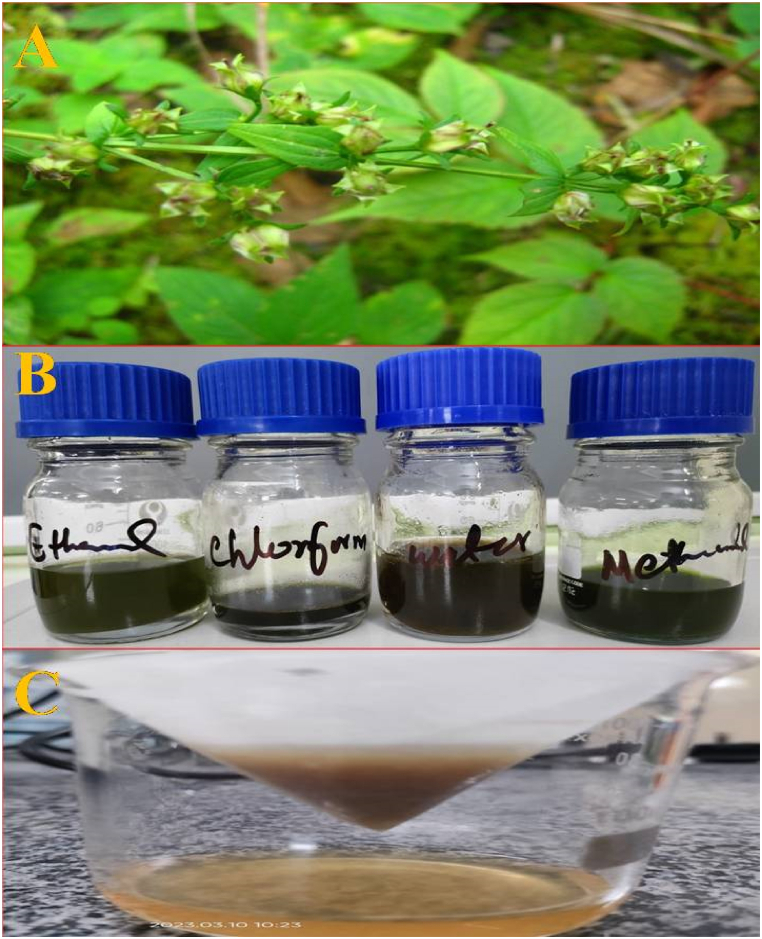
Preparation and filtration of different extracts of *Swertia chirata.* A: *Swertia chirata* plant. B: Various extracts of *Swertia chirata* including ethanolic, methanolic, and chloroform extracts. C: Filtration of different extracts of *Swertia chirata*.

To extract compounds from the powdered samples, the stem, and leaves were subjected to extraction using methanol, ethanol, chloroform, and distilled water in a soxhlet apparatus at 60 °C for 48 h. After extraction, the resulting extracts were allowed to cool at room temperature and filtered using Whatman No.1 filter paper and then evaporated to complete dryness using a flash evaporator [Fig. 2(A–C)]. The dried extracts were scraped and stored in a clean and sterile container for further use [25].

### 2.4 Phytochemical analysis

The qualitative phytochemical tests were conducted to detect the presence of different secondary metabolites.

### 2.5 Antioxidant activity

Antioxidant content was determined by using DPPH assay [26]. 1 mg of plant extract was mixed in 1 mL of DMSO to prepare the sample solution. From the above stock solution, a further 3 dilutions were made ranging from 25 to 100 μL. Then 50 μl of the sample solution was taken in the Eppendorf and mixed with 950 μl of methanol DPPH (0.004 %) was added in an Eppendorf tube to make the final volume of 1000 μl. Ascorbic acid was used as the positive control and DMSO as the negative control. Then the reaction mixture was kept in the incubator for 30 min in the dark at a room temperature of 25 °C. After that, the change in color of the mixture was observed. The appearance of yellow color indicates the presence of plant oxidation potential. The absorbance of the sample was measured by using a spectrophotometer at the wavelength of 517 nm.

### 2.6 Calculations

(A control - A sample) x 100/A control A = Absorbance.

### 2.7 Synthesis of silver nanoparticles

**Fig. 3:**
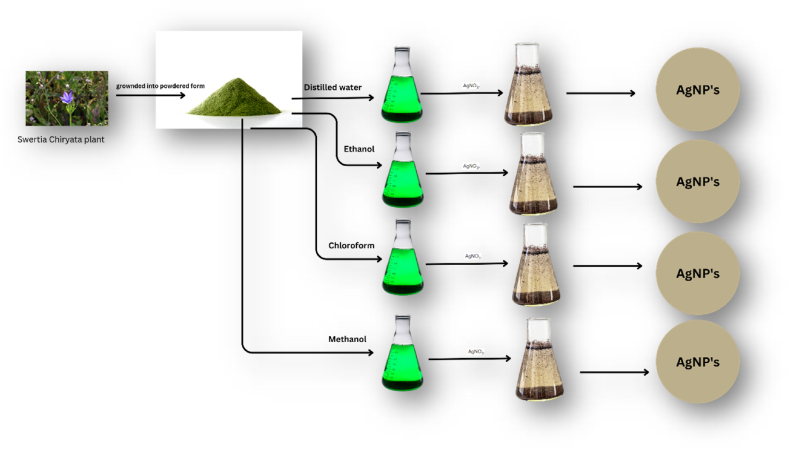
Siver Nanoparticles Synthesis from various extracts of *Swertia chirata*.

To synthesize silver nanoparticles (AgNPs) in an environmentally friendly manner, the *Swertia chirata* plant extracts were introduced into a solution of silver nitrate (AgNO3) and stirred continuously while maintaining an ambient temperature of 15 °C–25 °C [27]. The synthesis of AgNPs was observed at different intervals of time, with varying concentrations of both silver nitrate (AgNO3) and plant extracts being used in the process (Fig. 3).

### 2.8 Characterization of synthesized silver nanoparticles

To investigate the bio-reductive properties of *Swertia chirata* leaf extracts and their ability to form AgNPs, a BioBase BK-D590 double-beam UV–visible spectrophotometer with a wavelength range of 190 nm–1100 nm was employed [28]. Optical density (OD) measurements were recorded to confirm the reduction of silver nitrate during the process.

The elemental analysis of the green synthesized nanoparticles was performed with an EDX (model: OXFORD_INCA PENTx3) and recorded with an electronic microscope (model: JEOL JSM 5410).

The morphological characteristics of biosynthesized silver nanoparticles produced from plant extract have been studied using SEM. After adding AgNO3 for 48 h, the solutions were spread out on slides to create the SEM samples. To make the samples conductible, a thin coating of platinum was applied to them. Following that, the samples were characterized in the SEM using a 20 kV accelerating voltage.

### 2.9 Test microorganisms

The common human pathogenic strains such as *Escherichia coli*, *S. capitis*, *Pseudomonas aeruginosa,* and *B. subtilis* were obtained from the Department of Microbiology, Kohsar University Murree.

### 2.10 Assessment of antimicrobial activity of plant extracts and silver nanoparticles

To determine the antimicrobial effectiveness of silver nanoparticles and the plant extracts of *Swertia chirata* against bacterial strains such as *Escherichia coli,* and *S. capitis. Pseudomonas aeruginosa* and *B. subtilis*, the well-diffusion method was utilized [29]. Overnight cultures of each pathogenic strain were taken and 100 μl of bacteria were evenly spread on Muller Hinton Agar plates using a sterile glass spreader. Gel puncture was used to make 6 mm wells on the agar plates, and different concentrations (25, 50, and 100 μl) of silver nanoparticle and ethanolic, methanolic, chloroform, and aqueous extracts of the plant were poured into each well using a micropipette. The study aimed to investigate the antimicrobial activity of *Swertia chirata* plant extracts and silver nanoparticles prepared in four different solvents, namely ethanol, methanol, chloroform, and distilled water, against four pathogenic bacteria, *Escherichia coli, S. capitis, Pseudomonas aeruginosa,* and *B. subtilis*. Antibiotic discs such as cefixime and norfloxacin were used as positive controls. The plates were incubated at 37 °C for 24 h in an incubator for further growth. Following inoculation, the diameter of the zone of inhibition around each well was measured.

## 3 Results and discussion

### 3.1 Qualitative phytochemical analysis

*Swertia chirata* has great importance in traditional medicine as well as folk medicine, for the treatment of several diseases. The plant has several bioactive secondary metabolites that are good sources of medicines for curing numerous microbial diseases and improving the condition of human health [14,17]. At present, several researchers have led their attention to exploring various phytochemical constituents from this plant. The presence of a diversified group of pharmacologically bioactive chemicals belonging to several groups, such as xanthones and their derivatives, secoiridoids, lignans, iridoids, alkaloids, terpenoids, and flavonoids, are credited to *Swertia chirata*'s broad range of biological activities [14,30]. Other components include chitin, ophelic acid, palmitic acid, oleic acid, and stearic acid. Chiratanin, which is found in several parts of *Swertia chirata*, was the first dimeric xanthone to be isolated [31].

In the current study, the phytochemical analysis of *Swertia chirata* collected from the Murree Forest, Punjab was carried out on different extracts (ethanolic, methanolic, chloroform, and distilled water). In phytochemical analysis, qualitative and quantitative tests were performed. Qualitative tests were performed to know the presence or absence of the different compounds in these plant extracts. The extracts of *Swertia chirata* showed positive results for several phytochemicals. Ethanolic extract of *Swertia chirata* contained all the phytochemicals including phenols, flavonoids, alkaloids, terpenoids, tannins, saponins, glycosides, steroids, phytosterol, xanthones, coumarins, carbohydrates, anthocyanin, quinine, and lignin. Chloroform extract lacked phenols and coumarins while methanolic extract showed negative results for saponins and lignin (Table 1). Similar metabolites were also isolated from the *Swertia chirata* plant growing in Nepal [32,33], India [34], Pakistan [35], and China [13].

**Table 1:** Qualitative phytochemical analysis of different extracts of *Swertia chirata*.

Phytochemicals	Distilled water extract	Methanolic extract	Ethanolic extract	Chloroform extract
Phenols	+	+	++	–
Flavonoids	+	++	+	++
Alkaloids	+	+	+++	+
Terpenoids	++	++	+	++
Saponins	+	–	++	+
Tannins	+	+	++	+++
Glycosides	–	+++	+	++
Steroid	+	+	+	+
Phytosterol	–	++	+++	++
Xanthones	++	++	+	+
Coumarins	–	+	+	–
Carbohydrates	++	+	+++	++
Anthocyanin	–	+++	++	+++
Quinone	+	++	++	+
Lignin	+	–	+	++

### 3.2 Quantitative phytochemical analysis

Quantitative phytochemical analysis of a different extract of the plant was performed. In which the total phenolic content was expressed by using the standard curve equation (Y = 0.0038x+0.034), the slop was generated for x which is for Gallic Acid concentration 0.0038 and 0.0346 for the intersection. Total flavonoid content (TFC) was evaluated by using standard curve EQ(Y = 0.0038x+0.034), the slop was generated for x which is the quercetin concentration 0.0038, and 0.0346 is for the intersection. Numerous plants contain flavonoids, which are thought to decrease the cyclooxygenase enzyme's activity and have antibacterial and antiparasitic properties [36,37]. The results revealed that maximum TPC (197.64 ± 1.00 Gallic Acid Eq.mg/g) and TFC (149.58 ± 0.15 Quercetin Eq.mg/g) were recorded in methanolic extract of *Swertia chirata*, followed by ethanolic extract. Whereas the lowest TPC and TFC were expressed by aqueous extract (Table 2). In a previous study, the methanolic extract of the *Swertia chirata* plant collected from Nepal contained 26.16 ± 0.25 mg QE/g of TFC and 67.49 ± 0.50 GAE/g of TPC [38]. In the ethanol extract of *Swertia chirata*, Chen et al. [39] calculated the total flavonoid content to be 4.98 ± 0.40 mg rutin equivalents/g, and Tripathi et al. [40] found 10.6 μg equivalents of quercetin in 50 g of aqueous extract of *Swertia chirata*. Furthermore, differences in plant collection time and site may affect the concentrations of flavonoid and phenolic content [41].

**Table 2:** Qualitative phytochemical analysis of different extracts of *Swertia chirata*.

Extract	TPC(Gallic Acid Eq.mg/g)	TFC (Quercetin Eq.mg/g)
Ethanolic extract	192.64 ± 0.15	144.28 ± 0.40
Methanolic extract	197.64 ± 1.00	149.58 ± 0.15
Chloroform extract	155.71 ± 1.67	147.36 ± 0.95
Aqueous extract	147.56 ± 0.15	139.29 ± 0.80

### 3.3 Antioxidant potential of various extracts of *Swertia chirata*

Antioxidants play an important role in protecting against damage by reactive oxygen species. Antioxidants are biologically active phytochemicals that are naturally found in various fruits, vegetables, grains, and herbs which perform a protective function as an effective defense against oxidative damage from oxidizing agents and free radicals [37]. Cells have evolved a variety of defense systems based on both water-soluble and lipid-soluble antioxidants and antioxidant enzymes. A major proportion of antioxidant systems of the human body are dependent on dietary constituents. Antioxidants from plant extract are compounds that demonstrate biological activity that can protect the body from damage caused by free radical-induced oxidative stress [42,43].

The stable free radical DPPH may take an electron or a hydrogen atom to transform into a stable diamagnetic radical, which is then scavenged by a substrate that can donate protons. According to reports, the phenolic compounds can scavenge DPPH radicals which causes their absorbance to reduce [36,44]. This discoloration from purple to yellow is the consequence of the interplay between antioxidant molecules and radicals.

**Fig. 4:**
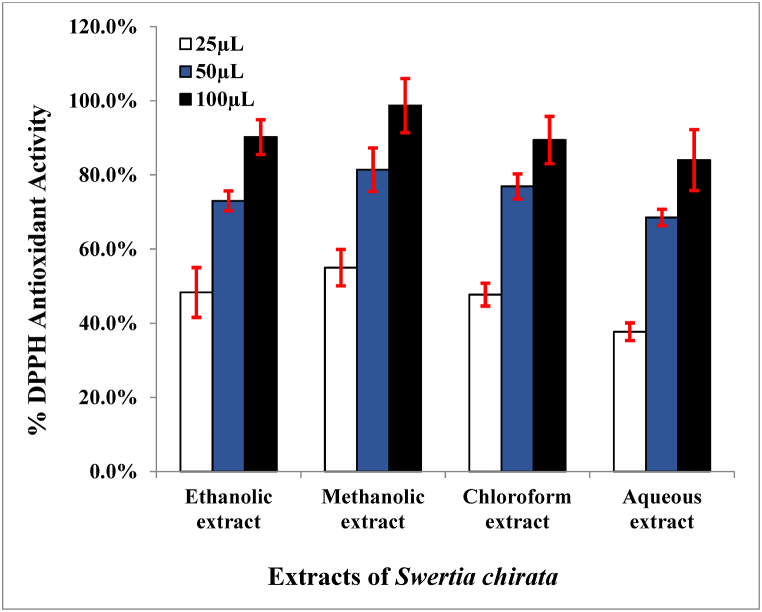
DPPH Antioxidant potential of different extracts of *Swertia chirata*.

All the extracts demonstrated concentration-dependent radical scavenging activity. The percent DPPH antioxidant activity increased with the increase in the concentration of extracts. At higher concentrations, all the extracts showed significant levels of antioxidant activity. The maximum antioxidant activity was recorded with 100 μl of methanolic extract (98 ± 7.3%), followed by ethanolic extract (90.2 ± 4.6%) at similar concentrations. The lowest percentage of antioxidant activity was shown by the aqueous extract (37.7 ± 2.4%) of *Swertia chirata* at 25 μl concentration (Fig. 4). Previously, 100 μg/mL of methanolic extract of *Swertia chirata* collected from the Galyat region of Khyber Pakhtunkhwa, Pakistan had shown 85.77 ± 2.49% antioxidant potential [41]. Sharma et al. [45] assessed the antioxidant activity of the *Swertia chirata* extract (collected from India) by measuring its free radical scavenging ability (DPPH test). The antioxidant activity of acetonic and methanolic plant extracts was investigated using butylated hydroxytoluene. The EC50 value of methanolic extract was determined to be 27.70 μg/mL, which was equivalent to the EC50 value of butylated hydroxytoluene extract (17.75 μg/mL). This demonstrates unequivocally that the methanolic extract of *Swertia chirata* has strong antioxidant properties. Similarly, Chen et al. [46] used antioxidant assays such as the reducing power and beta-carotene assay to examine the antioxidant properties of the 70% ethanolic extract of *Swertia chirata*. According to the findings, extracts made from 70% ethanol had a significant level of DPPH scavenging activity (IC50 = 267.80 μg/mL).

### 3.4 Biosynthesis and characterization of silver nanoparticles

In the current study, *Swertia chirata* plant extracts were used to biosynthesize silver nanoparticles. The Silver ions were converted to NPs with the presence of plant extract. A stabilizing and capping agent was provided as a plant extract. Numerous investigations have found that plant extract contains active substances that lead to the synthesis of AgNPs [44,47,48]. Furthermore, the environmentally friendly and toxin-free production of NPs utilizing plant component extract is of paramount relevance [48].

SEM, EDX, and UV–visible spectroscopy were employed to analyze the newly phytosynthesized AgNPs. Combinations of techniques are often required for NP characterization since a single methodology is inadequate to accurately describe the colloidal NPs. Several characterizing peaks are seen for the synthesis of AgNPs, mainly in the range of 410–480 nm [[49], [50], [51], [52]]. However, colloidal AgNPs may have varied sizes and morphologies at different wavelengths.

**Fig. 5:**
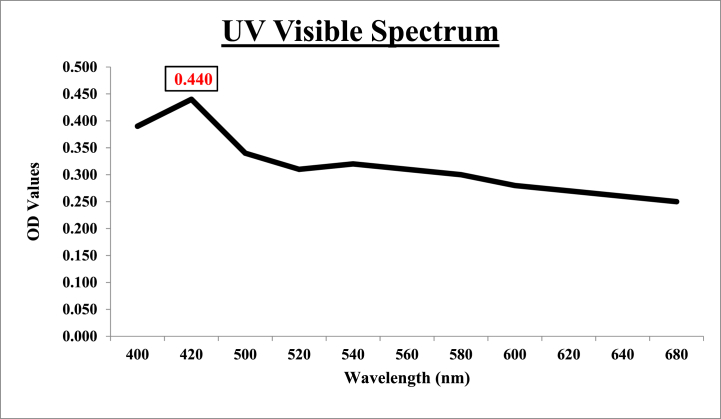
UV–Vis spectra of photosynthesized silver nanoparticles using extract of *Swertia chirata*.

It was reported previously that the SPR peak of silver nanoparticles synthesized using *Swertia chirata* was detected at 450 nm [53]. In the present study, silver nanoparticles were characterized using a double-beam photo spectrometer ranging in wavelength from 400 nm to 600 nm. The highest OD value was observed at the wavelengths of 400 nm and 420 nm (0.390 and 0.440, respectively). Color changes of silver nanoparticles were observed at different wavelengths from dark brown to red. A characterization peak for the AgNPs made from *Swertia chirata* can be seen in Fig. 5.

**Fig. 6:**
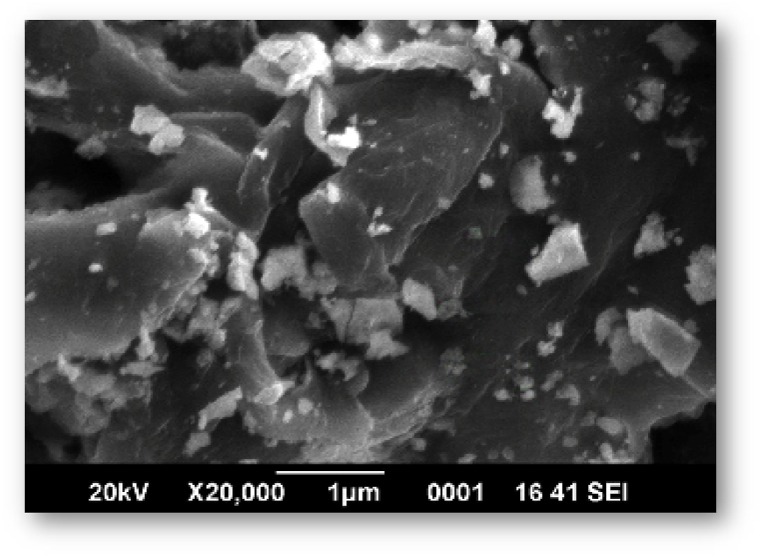
SEM of silver nanoparticles green synthesizing through *Swertia chirata*.

The structural characteristics of the produced AgNPs were examined using SEM. According to SEM examination, the newly photosynthesized nanoparticles were round and cuboidal in form, with a diameter ranging from 80 to 110 nm (Fig. 6). Similar SEM results were also reported in a previous study where aqueous extract of *Swertia chirata* (from India) was used to synthesize silver nanoparticles [53].

**Fig. 7:**
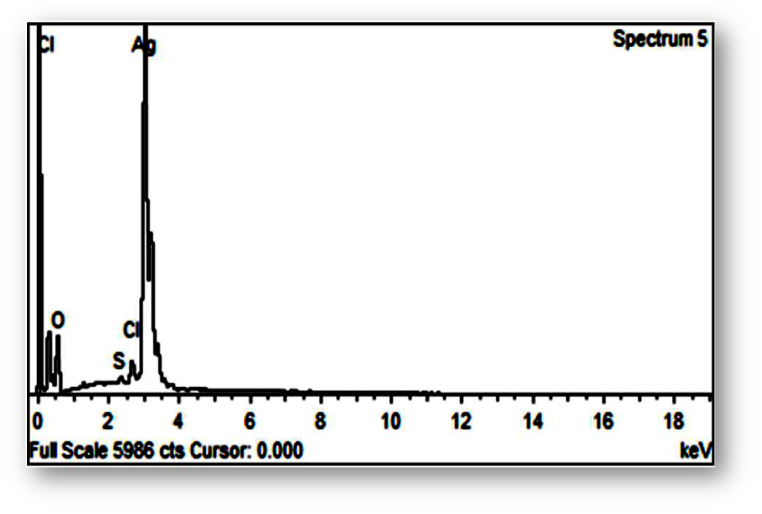
EDX spectrum of photosynthesized silver nanoparticles through *Swertia chirata*.

EDX was used to conduct an elemental analysis of the produced AgNPs. Using an EDX detector, silver ions were confirmed to be present. The most prominent silver peak was visible in the 2.5–3.5 keV range of the EX-spectrum (Fig. 7). The silver atoms in the nanoparticles produce significant signals at 3 keV, a peak that is typical of metallic silver nanocrystals. The findings were consistent with other studies of silver nanoparticle EDX spectra [[54], [55], [56]].

### 3.5 Antimicrobial susceptibility test

The problem of drug resistance in bacterial infections is a major challenge in modern medicine [57]. Antibiotic overuse and misuse have led to the emergence of drug-resistant strains, making traditional antibiotics less effective [[58], [59], [60]]. Therefore, there is a growing interest in the development of alternative treatments, such as plant-based antimicrobials [61]. This study investigated the antimicrobial activity of *Swertia chirata* plant extracts and silver nanoparticles against human pathogenic bacteria, including *Escherichia coli, S. capitis, Pseudomonas aeruginosa*, and *B. subtilis*. These pathogenic microorganisms not only cause disease in humans but also food-borne pathogens and play a vital role in food spoilage [62].

**Fig. 8:**
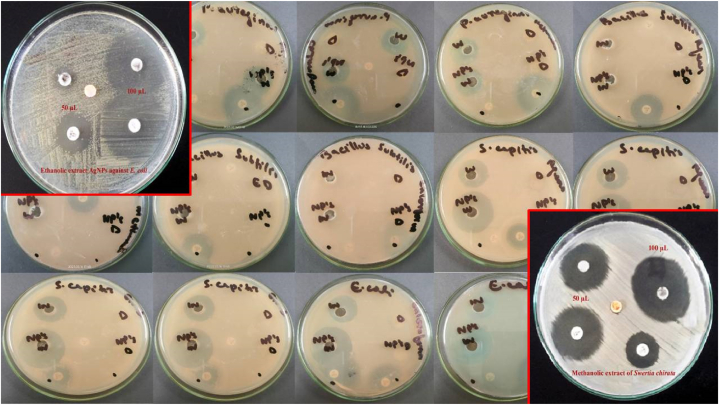
Antibacterial activity of different plant extracts, nanoparticles, and antibiotics against various bacteria.

In the antimicrobial susceptibility test, the zones of inhibition were recorded in mm diameter. The plant extract prepared in all four solvents and their silver nanoparticles and norfloxacin antibiotic discs showed significant antibacterial activity against the pathogenic microbes (Table 3, Fig. 8). The zone of bacterial growth inhibition increased with the increase in concentration of extracts and nanoparticles. The results showed that *Swertia chirata* plant extracts and silver nanoparticles had significant antimicrobial activity against *Escherichia coli, S. capitis, B. subtilis,* and *Pseudomonas aeruginosa*. However, the antibiotics applied to the microbes showed limited antibacterial activity, indicating that these bacteria are resistant to antibiotics. In the current study, significant antibacterial activity was detected against *E.coli* (30.7 ± 2.63 mm), followed by *S. capitas* (29.3 ± 1.69 mm) and *Pseudomonas aeruginosa* (28.8 ± 1.37 mm) when exposed to higher concentration (100 μl) of methanolic extract of *Swertia chirata* or AgNps synthesized from the same extract. A moderate level of antibacterial activity was also exhibited by the ethanolic, chloroform, and aqueous extracts of *Swertia chirata*. A previous study conducted in India reported that AgNPs synthesized from methanolic extract of *Swertia chirata* leaves exhibited significant antibacterial activity against *Escherichia coli* and *Klebsiella pneumonia* [63].

**Table 3:** Antibacterial activity of plant extracts, silver nanoparticles, and antibiotics against *E.coli, S. capital, Pseudomonas aeruginosa,* and *B. subtilis* (Zone of inhibition is recorded as mm).

Extracts	*E. coli*	*S. capital*	*P. aeruginosa*	*B. subtilis*
25 μl concentration				
Ethanolic extract	5.1 ± 0.18	7.3 ± 1.02	7.9 ± 1.17	2.4 ± 0.82
Ethanolic nanoparticles	7.9 ± 0.24	8.8 ± 0.61	9.3 ± 1.07	5.1 ± 0.21
Methanolic extracts	6.2 ± 0.21	4.7 ± 0.21	6.9 ± 0.63	1.3 ± 0.11
Methanolic extracts nanoparticles	10.7 ± 0.46	12.8 ± 0.91	9.6 ± 0.69	6.6 ± 1.08
Chloroform extract	8.7 ± 0.17	7.7 ± 0.58	6.3 ± 0.46	1.9 ± 0.79
Chloroform nanoparticles	10.6 ± 0.82	10.3 ± 0.52	7.6 ± 0.85	3.9 ± 0.61
Aqueous extracts	4.1 ± 0.43	6.2 ± 0.73	4.9 ± 0.31	2.7 ± 0.43
Aqueous nanoparticles	7.2 ± 0.69	7.9 ± 0.48	5.1 ± 0.42	4.4 ± 0.77
Cefixime antibiotic	1.21 ± 0.08	4.8 ± 0.66	3.3 ± 0.29	0 ± 0.00
Norfloxacin antibiotic	6.3 ± 0.12	5.5 ± 0.39	4.1 ± 0.30	0.3 ± 0.02
50 μl concentration				
Ethanolic extract	6.9 ± 0.88	9.6 ± 1.22	8.5 ± 0.97	4.3 ± 0.62
Ethanolic nanoparticles	10.2 ± 1.05	11.7 ± 0.98	10.6 ± 0.77	5.6 ± 0.44
Methanolic extracts	7.9 ± 0.48	7.6 ± 0.77	8.3 ± 1.19	3.3 ± 0.27
Methanolic extracts nanoparticles	12.6 ± 0.63	14.1 ± 1.55	11.2 ± 1.91	7.7 ± 0.88
Chloroform extract	9.4 ± 1.02	8.2 ± 0.76	8.1 ± 0.67	5.7 ± 0.82
Chloroform nanoparticles	13.1 ± 1.25	12.9 ± 0.91	8.9 ± 0.54	7.0 ± 0.48
Aqueous extracts	6.4 ± 1.03	7.9 ± 1.62	6.6 ± 0.53	4.1 ± 0.63
Aqueous nanoparticles	8.9 ± 0.88	10.4 ± 1.19	9.2 ± 1.12	4.4 ± 0.68
Cefixime antibiotic	3.7 ± 0.61	5.8 ± 0.73	4.7 ± 0.59	0.7 ± 0.08
Norfloxacin antibiotic	8.4 ± 0.89	7.5 ± 0.92	5.8 ± 0.49	1.5 ± 0.16
100 μl concentration				
Ethanolic extract	18.3 ± 1.71	18.2 ± 0.23	16.9 ± 0.63	4.4 ± 0.46
Ethanolic nanoparticles	25.9 ± 1.04	24.8 ± 0.63	22.6 ± 1.09	12.3 ± 0.79
Methanolic extracts	25.1 ± 0.98	29.3 ± 1.69	21.0 ± 1.77	6.4 ± 1.30
Methanolic extracts nanoparticles	30.7 ± 2.63	28.7 ± 2.01	28.8 ± 1.37	14.9 ± 1.21
Chloroform extract	24.0 ± 0.77	21.3 ± 0.48	21.3 ± 0.66	5.3 ± 0.79
Chloroform nanoparticles	26.7 ± 0.91	24.1 ± 0.61	20.1 ± 1.15	9.1 ± 1.21
Aqueous extracts	22.1 ± 0.83	17.2 ± 1.31	17.9 ± 0.61	4.1 ± 0.36
Aqueous nanoparticles	27.2 ± 1.29	26.5 ± 0.78	22.1 ± 0.88	6.7 ± 0.48
Cefixime antibiotic	5.2 ± 0.44	8.8 ± 0.56	11.3 ± 0.29	1.9 ± 0.14
Norfloxacin antibiotic	21.3 ± 0.78	19.3 ± 0.89	23.7 ± 1.10	4.2 ± 0.42

According to Ahluwalia et al. [64], 20 μg/mL AgNPs synthesized from an aqueous extract of *Swertia paniculata* showed significant antibacterial activity against *P. aeuriginosa* with a zone of inhibition of 18.80 ± 0.72 mm, while *Staphylococcus aureus* exhibited the zone of inhibition of 14.67 ± 0.35 mm using. It was also observed that gram-negative bacterial strains, such as *Pseudomonas aeruginosa* and *Klebsiella pneumonia* were more susceptible to AgNPs than *Staphylococcus aureus* [64]. Similar results were also observed in the current study, *B. subtilis* showed remarkable resistance against AgNPs and plant extracts as compared to other microbes. No zone of inhibition was formed when *B. subtilis* was treated with cefixime at a lower concentration (25 μl) (Table 3).

The higher antimicrobial activity of ethanolic and methanolic extracts and silver nanoparticles can be attributed to the presence of phytochemicals such as alkaloids, flavonoids, and tannins, which are known for their antibacterial activity [65]. Gram-positive bacteria have thick peptidoglycan layers in their cell walls, whereas gram-negative bacteria have thin peptidoglycan layers. The degree of bioactivity is significantly influenced by how permeable a chemical is to the peptidoglycan layer [66,67]. Due to the compositional differences between the cell walls of Gram-positive and Gram-negative bacteria, several antimicrobial agents exhibit varying behaviors [68]. Numerous researchers have put up various hypotheses.

According to Sondi et al. [69], the interaction between the silver cations generated by NPs and the bacterial cell wall enhanced the permeability of the cell membrane, which would explain the significant bactericidal activity. The attachment of silver NPs or ions to the cell wall results in a concentration of envelop protein precursors, which denaturizes the protein [70]. According to literature studies, when silver ions interact with the thiol groups of enzymes, Reactive Oxygen Species (ROS) are produced, which causes the cell wall to self-destruct [71]. According to Morones et al. [72], silver works as a mild acid, causing the DNA to be damaged along with its nuclear machinery. The increased activity of the AgNPs may be attributed to their nanosize and phytochemical capping. Due to their nano size, silver nanoparticles may have a greater penetration rate in cell walls, and phytochemicals can improve their biological activities [73]. Dihydrofolate reductase (DHFR), an essential cofactor involved in the production of amino acids, may be bound by phytochemicals [74].

In conclusion, the findings of this study suggest that *Swertia chirata* plant extracts and silver nanoparticles can be used as potential antibacterial agents against pathogenic bacteria. However, further studies are needed to investigate the mechanism of action and toxicity of these extracts and nanoparticles before they can be considered for clinical use.

## 4 Conclusion

In the current study, phytochemical analysis of the medicinal plant collected for the first time from the forests of Murree, Punjab, Pakistan (*Swertia chirata*) revealed that it contains several phytochemicals with significant antioxidant potential. The current work also illustrated a green and environmentally acceptable nano-synthetic mechanism for the photoproduction of durable and spherical nanosilver particles. During the photo-manufacturing of silver nanoparticles (AgNPs) from silver nitrate, different extracts of the medicinal plant *Swertia chirata* (aqueous, ethanolic, methanolic, and chloroform extracts) were used as a capping and reducing agent. The findings of physical-chemical characterization showed that the produced AgNPs had a spherical form and the optimal size range. Furthermore, the result of antibacterial analysis suggests that the methanolic and ethanolic extracts and silver nanoparticles could be used as natural alternatives to synthetic antibiotics for the treatment of bacterial infections, particularly against *E. coli, P. aeruginosa, B. subtilis,* and *S. capitis*. Overall, this environmentally friendly technology may be a competitive alternative to existing physical or chemical processes for the production of nanoparticles and may be employed as inhibitors in a range of biological applications. Further studies are needed to identify the active compounds responsible for the antimicrobial activity and to evaluate the toxicity of the plant extracts and silver nanoparticles.

## Data availability statment

No data was used for the research described in the article.

## CRediT authorship contribution statement

**Muhammad Adnan Shereen:** Methodology, Investigation, Formal analysis. **Aftab Ahmad:** Formal analysis, Data curation. **Hashir Khan:** Methodology, Investigation, Formal analysis, Data curation. **Sadia Mehmood Satti:** Visualization, Formal analysis, Data curation, Conceptualization. **Abeer Kazmi:** Writing – original draft, Supervision, Conceptualization. **Nadia Bashir:** Writing – review & editing, Writing – original draft, Formal analysis, Data curation. **Muhammad Shehroz:** Software, Resources, Methodology, Formal analysis. **Shahid Hussain:** Writing – original draft, Software, Resources, Methodology, Investigation, Formal analysis, Data curation. **Muhammad Ilyas:** Software, Resources, Methodology, Investigation, Formal analysis, Data curation, Conceptualization. **M. Ijaz Khan:** Writing – original draft, Supervision, Project administration, Investigation, Data curation, Conceptualization. **Hatoon A. Niyazi:** Writing – review & editing, Visualization, Validation, Supervision, Project administration, Investigation, Funding acquisition, Formal analysis, Conceptualization. **Ferjeni Zouidi:** Writing – review & editing, Visualization, Validation, Supervision, Resources, Project administration, Investigation, Funding acquisition, Formal analysis, Data curation, Conceptualization.

## Declaration of competing interest

The authors declare that they have no known competing financial interests or personal relationships that could have appeared to influence the work reported in this paper.
